# Body composition, anthropometry, and resting energy expenditure in adults with achondroplasia: a pilot study to determine best practices

**DOI:** 10.1186/s13023-025-03912-z

**Published:** 2025-10-21

**Authors:** Kerry Schulze, John McGready, Bobbie Barron, Celide Koerner, Julie Hoover-Fong

**Affiliations:** 1 Program in Human Nutrition, Department of International Health, Johns Hopkins University Bloomberg School of Public Health, Baltimore, MD USA; 2 Department of Biostatistics, Johns Hopkins University Bloomberg School of Public Health, Baltimore, MD USA; 3 Institute for Clinical and Translational Research, Johns Hopkins University School of Medicine, Baltimore, MD USA; 4 Greenberg Center for Skeletal Dysplasias, McKusick-Nathans Department of Genetic Medicine, Johns Hopkins University School of Medicine, Baltimore, MD USA

**Keywords:** Achondroplasia, Body composition, Obesity, Adiposity, Fat, Percent body fat, Fat free mass, Resting energy expenditure

## Abstract

**Background:**

To guide recommendations for preferable body weights and energy requirements in those with achondroplasia, particularly given concern for overweight and obesity, studies of body composition, energy utilization, and health outcomes must be conducted in those with this rare (1 in 20,000–30,000 births) condition. We compared body composition using bioelectrical impedance analysis (BIA) and anthropometry against dual energy X-ray absorptiometry (DXA) as a gold standard and characterized resting energy expenditure (REE) among adults with achondroplasia in a pilot study of the feasibility and validity of these approaches and describe implications of their use in this population.

**Methods:**

Twenty adults (9 female, 38.5 ± 6.3 years; 11 male, 35.1 ± 10.4 years) with achondroplasia were recruited for a week-long study with an overnight inpatient component to assess the feasibility of conducting tests of health, physical activity, and function. Body composition was assessed using anthropometry, tetrapolar BIA, and DXA under standardized conditions. REE was assessed in the morning in a fasted, resting state and expressed relative to whole body mass and fat free mass. Aspects of body composition were compared by sex using t-tests, and total body fat, percent body fat, and fat free mass by BIA were compared to DXA by linear regression and Bland–Altman analysis.

**Results:**

Height, weight (although ~ 9 kg greater in males), fat free mass (FFM; by DXA or BIA), waist, hip, arm and neck circumference, and REE did not differ by sex; body mass index, total and percent body fat, triceps, subscapular, and arm fat area were greater in females while arm muscle area was greater in males. By DXA, fatness of the extremities was greater in females. Estimates of total and percent body fat were similar by DXA and BIA in both sexes combined and were strongly related to waist circumference.

**Conclusions:**

In achondroplasia, BIA and key anthropometric indicators could assess excess adiposity. We found REE to be appropriate for body size, suggesting that weight control might be dependent on improved fitness and activity levels in this population. Comprehensive assessment of body composition in relation to health outcomes in a larger achondroplasia cohort is required.

**Supplementary Information:**

The online version contains supplementary material available at 10.1186/s13023-025-03912-z.

## Background

Achondroplasia is the most common short stature skeletal dysplasia, affecting 1 in 20,000–30,000 births [1–4]. Physical characteristics of achondroplasia include shortened limbs, underdevelopment of the mid-face region, macrocephaly, and lordosis. Adult height is approximately 4′1″ in females and 4′3″ in males [5–7]. Short stature in patients with achondroplasia leaves a smaller frame on which body weight can be distributed, which, along with limited mobility due to pain and other factors [8–11], may predispose to obesity. In fact, deaths due to cardiovascular disease occur at rates 10 times higher among those with achondroplasia compared to average stature adults [12], and hypertension is prevalent [13–15], suggesting consequences of excess adiposity.

Despite this concern, condition-specific criteria for diagnosing obesity have not been defined. Conventional cutoffs to define overweight and obesity using body mass index (BMI, kg/m^2^) in the average stature population are not appropriate for those with achondroplasia [16]. Moreover, the unique body proportions of achondroplasia challenge assumptions inherent in body composition assessment techniques that are commonly used among average stature populations to distinguish fat and fat free mass compartments, including prediction equations that utilize anthropometric measures and bioelectrical impedance analysis (BIA) [17, 18]. Fredwall et al. [19] successfully utilized novel magnetic resonance imaging (MRI) methods to quantify lean and fat mass in a cohort of individuals with achondroplasia, but this assessment requires access to MRI instrumentation with specific software. Dual energy x-ray absorptiometry (DXA) may be more readily accessible for assessing body composition in this population but currently is not widely applied in the clinical setting.

Energy requirements are also not well characterized in those with achondroplasia. Resting Energy Expenditure (REE) is typically the major contributor to energy utilization, while the contribution of physical activity, which is often limited in individuals with achondroplasia [20], is most variable. Low voluntary energy expenditure can contribute to undesired fat gain and lower muscle mass. Because of its metabolic activity, lean (i.e., muscle) mass is more strongly associated with REE than fat, consistent with potential increases in REE and total energy utilization with exercise, among other health benefits [21, 22]. However, while linked with the metabolic activity of lean tissue, REE also increases in proportion to body size: REE in average stature populations increases across obesity categories even as lean mass differs minimally [23, 24]. Thus, estimating energy requirements for optimal health requires understanding the contribution of lean relative to total body mass to REE in people with achondroplasia.

To guide recommendations for preferable body weights and energy requirements in those with skeletal dysplasias, comprehensive studies of body composition and energy utilization must be conducted and related to health outcomes. Yet, there is little guidance on appropriate methods for conducting these types of assessments in those with achondroplasia. In this paper we provide data from a comprehensive cross-sectional pilot study of 20 adults with achondroplasia. Here we examine body composition with anthropometric measures and bioelectrical impedance analysis, using DXA as a gold standard. Additionally, we explore the association of body composition with resting energy expenditure to ascertain feasibility, validity, and implications of using these measurements.

## Methods

### Population

Individuals with achondroplasia from the mid-Atlantic region were recruited to participate in an overnight pilot study at Johns Hopkins Hospital (JHH) that involved a variety of approaches for assessing aspects of body composition, REE, physical stamina and activity, and included sleep characteristics, bloodwork, and echocardiography, among other tests. Participants were followed for a week following their discharge to collect additional information on accelerometry, self-reported physical activity, and diet. Given the level of commitment required, participants were recruited based on direct contact with local clinic patients, word of mouth, and advertisements at regional Little People of America (LPA) events; individuals with reasonable access to JHH were prioritized for recruitment. The intent was to study the acceptability of the methods to the participant population and ascertain the quality of data obtained. The study was approved by the JHH Institutional Review Board, and signed informed consent was obtained from each participant.

### Measures

Anthropometric measures including height and weight; circumferences of the waist at the most minimal point and at the umbilicus, hip, mid-upper arms, and neck; and triceps and subscapular skinfolds were measured. Anthropometry was done by clinical experts, with modifications to conventional techniques as required. For example, given the rhizomelia of the arms, the mid-upper arm landmark was a challenge to isolate for the circumference and skinfold measures. For standing height 2 contact points were required (i.e. occiput and buttock), which is a modification from the 4 (the addition of shoulders and heels) that are typically considered when measuring someone of average stature; specific protocols are required for clinical care and research of individuals with short stature, such that a separate manuscript devoted to modifications to anthropometric measures in this population was written [25]. Derived measures included BMI and mid-upper arm fat (AFA) and muscle (AMA) areas.

Tetrapolar (electrode placement on hands and feet of one side of the body), single frequency bioelectrical impedance analysis (BIA; Quantum X, RJL Systems) was conducted with participants in a supine position. Resistance measures were used in body composition equations developed by Sun et al. [26] for use in the general US population, given a lack of condition-specific equations for individuals with achondroplasia, to calculate fat free mass (FFM). Fat mass was calculated by difference of FFM from total body weight and expressed as a percent of total body weight. Prediction equations for body composition by BIA take account of body length and volume [17, 18], relative proportions of which are different in the achondroplasia population compared with those of average height. We therefore sought to establish the validity of this measure for assessing body composition in these study subjects relative to measures from dual energy x-ray absorptiometry (DXA) as a gold standard. Body composition by DXA was assessed using a Hologic QDR 4500. Fat free mass was calculated as the sum of lean mass and bone mass per body region. Fat mass for the regions of the body, in total, and as percent of total body weight was also summarized.

Resting energy expenditure (REE) was measured in the morning in a waking but fasted and restful state using a Vmax 229N indirect hood calorimeter (VIASYS Respiratory Care, Inc., Yorba Linda, CA). Data were obtained for at least 20 but not longer than 30 min, and energy expenditure under that condition was extrapolated to a 24-h period. To determine the comparability of REE per unit body weight with data from average stature populations, REE was expressed per total body weight and fat free mass.

### Data analysis

Because this was a pilot study, sample size goals were not set based on power calculations but rather on feasibility. Upon exploratory analysis of the findings, differences in parameters of interest by sex became obvious. Thus, t-tests were used to compare anthropometry, body composition, and REE outcomes by sex. Due to small sample size, findings were confirmed by non-parametric Wilcoxon ranksum tests. However, results of parametric tests are reported as no assumptions were violated and for ease of interpretation of regression results.

To examine the utility of using BIA to assess body composition, using DXA as the gold standard, linear regression was used to describe relationships between total fat mass, fat as percent of total body weight, and fat free mass between the two measurement types. Bland–Altman plots and a bias assessment was done, and limits of agreement reported. To determine the utility of BMI as a proxy measure for body fatness, we used linear regression with total and percent body fat from the gold standard DXA measure as the outcome with BMI as an explanatory variable, and we also examined FFM as an outcome relative to BMI to contrast findings with the measures of adiposity. Correlations between anthropometric measures and body composition by DXA by sex were also explored to ascertain the utility of using anthropometry as a proxy for distinguishing adiposity and FFM. Finally, we expressed REE per unit FFM and per total body weight and also explored the association of REE with these body composition parameters with linear regression. In all regression models, sex was examined as an independent variable and in an interaction term to determine if intercepts and/or slopes of associations differed by sex, respectively. Interactions at P < 0.20 were considered important, at which point the associations were stratified by sex. Otherwise, P < 0.05 was considered statistically significant. All statistical analyses were conducted using Stata 18.0 (StataCorp LLC, College Station, TX, USA).

## Results

Characteristics of study participants are shown in Table 1. Among 9 female and 11 male participants, the average age was 36.6 ± 8.7 and did not differ by sex. Weight and height did not differ statistically by sex, although BMI was higher among women. Based on measures from DXA, fat free mass did not differ between males and females, but total fat mass and percent body fat were nearly double in women compared to men. Fat mass differences by sex were greater in the extremities than in the trunk area. Findings for total fat free mass, fat mass, and percent body fat by BIA differed similarly by sex to those observed by DXA.

**Table 1:** Age, anthropometric, body composition, and resting energy expenditure among participants with achondroplasia

	Female (n = 9)	Male (n = 11)	P-value for difference^1^
Age, y	38.5 ± 6.3	35.1 ± 10.4	0.40
Weight, kg	60.0 ± 11.2	51.1 ± 11.7	0.10
Height, cm	123.5 ± 4.7	125.9 ± 5.2	0.30
BMI, kg/m^2^	39.0 ± 5.1	32.4 ± 8.1	0.05
*DXA measures*			
Total FFM, kg	33.6 ± 4.4	36.9 ± 4.9	0.1
Total fat mass, kg	25.7 ± 7.4	13.7 ± 8.1	0.003
Body fat, %	42.7 ± 5.2	25.2 ± 10.5	0.0003
*Regional body fat, kg*			
Trunk	12.1 ± 4.4	7.1 ± 5.0	0.03
Arms	3.3 ± 1.1	1.6 ± 0.9	0.001
Legs	9.4 ± 2.1	3.9 ± 2.5	< 0.0001
*BIA measures*			
Total FFM, kg	35.1 ± 4.2	37.9 ± 5.2	0.2
Total fat mass, kg	24.9 ± 7.3	13.3 ± 8.3	0.004
Body fat, %	40.7 ± 5.3	24.1 ± 11.1	0.0006
*Other Anthropometry*			
Waist circumference, minimal, cm	83.3 ± 9.4	79.5 ± 14.0	0.49
Waist circumference, umbilicus, cm	88.5 ± 11.5	81.3 ± 15.6	0.27
Hip circumference, cm	109.6 ± 9.3	91.9 ± 10.9	0.0012
Neck circumference, cm	36.4 ± 4.2	38.3 ± 3.8	0.31
Arm circumference (right), cm	32.7 ± 4.6	28.8 ± 4.6	0.072
TSF (right), mm	37.4 ± 9.6	16.4 ± 10.2	0.0002
SSF (right), mm	24.3 ± 7.7	15.7 ± 8.0	0.044
AFA (right), cm^2^	51.1 ± 17.0	22.4 ± 15.3	0.0009
AMA (right), cm^2^	35.7 ± 9.4	45.0 ± 10.0	0.049
Resting energy expenditure (REE), kcal/d	1266 ± 102	1256 ± 172	0.9
REE per FFM, kcal/kg/d	38.1 ± 3.4	34.4 ± 5.3	0.09
REE per body mass, kcal/kg/d	21.6 ± 3.3	25.8 ± 7.0	0.12

*BMI* body mass index, *DXA* dual energy x-ray absorptiometry, *FFM* fat free mass, *TSF* triceps skinfold, *SSF* subscapular skinfold, *AFA* arm fat area, *AMA* arm muscle area, *REE* resting energy expenditure

^1^By t-test

Anthropometric measurements tended to be greater among women for all but neck circumference and AMA (Table 1), but were only significantly so for hip circumference, triceps (TSF) and subscapular (SSF) skinfold measures, and AFA. Even at its minimum girth, waist circumference was above recommended cutoffs related to risk of metabolic syndrome of 80 cm in 7/9 women, compared to 1/11 men above the cutoff of 94 cm [27]. Arm muscle area was significantly lower in women.

Despite differences in body composition parameters, REE did not differ between males and females (Table 1). In women, REE per FFM was somewhat higher and per total body weight somewhat lower than in men, although neither was statistically significant. Simple and sex-adjusted linear regression did not reveal an association between REE and total body weight, with a 2.4 kcal (95%CI -4.0, 8.8) difference in REE per one kilogram increase in body weight (p = 0.44) in the sex-adjusted model. However, REE was positively associated with FFM in a sex-adjusted regression model, with a 14.5 (0.5, 28.6; p = 0.04) kcal increase in REE per one kilogram increase in FFM (not shown).

**Fig. 1:**
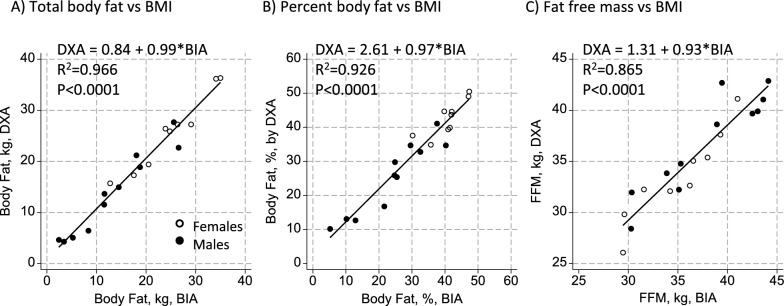
Associations between body composition parameters by dual energy X-ray absorptiometry (DXA) and Bioelectrical Impedance Analysis (BIA). Plots show associations of **A** total body fat, **B** percent body fat, and **C** fat free mass measured among adult females (n = 9) and males (n = 11) with achondroplasia using simple linear regression, along with the best fit line for describing the association between body composition by DXA and BIA-based measures. The slope and location of the line did not differ by sex, and the equations are shown on each graph

Compared to DXA as the gold standard measure of body composition in this study, BIA using the equation by Sun et al. [26] performed notably well at estimating total body fat, percent body fat, and FFM among men and women as shown in Fig. 1. Lines, including both locations and slopes, did not differ by sex. A one kilogram increase in body fat estimated by BIA was associated with a 0.99 (0.90, 1.08) kilogram increase in body fat by DXA, with 96.6% of the variance in body fat by DXA explained by BIA. For percent body fat, there was a 0.97 (0.83, 1.10)% increase in body fat by DXA per 1.00% increase in body fat estimated by BIA, with 92.6% of the variance in percent body fat by DXA explained. Finally, there was a 0.93 (0.75, 1.11) kilogram increase in fat free mass by DXA per one kilogram increase in FFM estimated by BIA, with 86.5% of the variance explained. Bland–Altman plots are shown in the Additional File (Supplementary Figs. 1–3) and major parameters of interest shown in Table 2. The findings show body fat to have been about 0.5 kg, or ~ 1% body weight, lower by BIA than DXA, and FFM to be just over one kilogram higher by BIA than DXA, although all consistent with zero bias. At the extremes of the distribution (95th percentiles), body fat would (at the y-intercept of 0 kg fat)  have been underestimated by 3.5 or overestimated by 2.5 kg in an individual, corresponding to underestimates of nearly 10% body fat to an overestimate of 7%. The lower limit of agreement at the 95th percentile was consistent with an underestimate of FFM of just over three kilograms, with an overestimate of nearly six kilograms at the upper limit of agreement.

**Table 2:** Bland–Altman parameters (at y-intercept of 0 kg or %) for comparisons of total body fat, percent body fat, and total fat free mass by BIA relative to DXA

Comparison	Bias (95% CI)	Lower to upper limit of agreement, 95%-ile
Body fat, kg	− 0.51 (− 2.47, 1.44)	− 3.54, 2.51
Body fat, %	− 1.4 (− 6.1, 3.3)	− 9.8, 7.0
FFM, kg	1.26 (− 5.60, 8.11)	− 3.37, 5.89

**Fig. 2:**
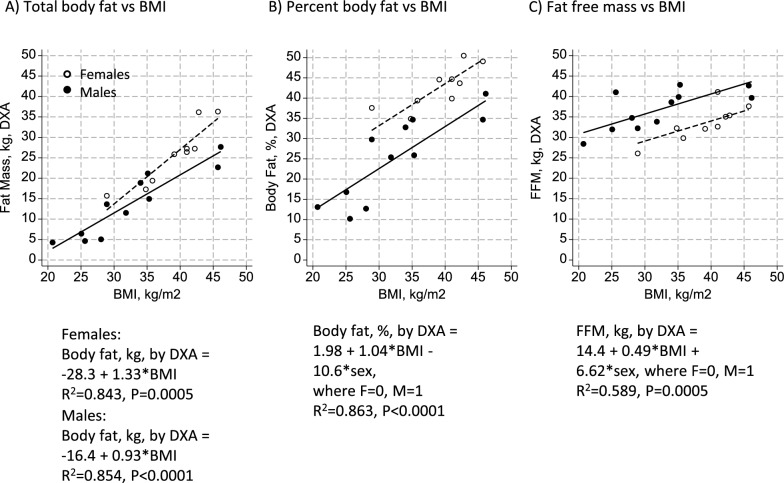
Associations between body composition parameters by dual energy X-ray absorptiometry (DXA) and Body Mass Index (BMI). Plots show associations of **A** total body fat, **B** percent body fat, and **C** fat free mass (FFM) among adult females (n = 9) and males (n = 11) with achondroplasia. The association of BMI with total body fat was steeper for females than males, as indicated in panel A. Percent body fat was 10.6% higher among women for a given BMI, and FFM was 6.62 kg lower in females for a given BMI, although the slopes in panels B and C were similar between sexes

BMI was associated with total body fat among both men and women (Fig. 2), although the slope of the association was greater among women, with a 1.33 (0.82, 1.85) kilogram increment in body fat per one unit increase in BMI in women, with 84.3% of the variance in total fat explained, compared to a 0.93 (0.64, 1.22) kilogram gain in total body fat per one unit increase in BMI in men, with a similar 85.4% of the variance in total fat explained. When expressed as percent body fat, the slopes of the association between BMI and percent body fat were similar between men and women with a 1.04 (0.70, 1.39)% increase per one unit gain in BMI, although women averaged 10.6 (5.5, 15.7)% more total body fat for a given BMI than men (P < 0.0001 for both sex and BMI), and with 86.3% of the variance in percent body fat explained among men and women combined. For FFM, a much lower 58.9% of the variance was explained by BMI among females and males combined, and FFM averaged 6.62 (3.12, 10.11) kilograms lower in women for a given BMI. A one unit increment in BMI was associated with a 0.49 (0.26, 0.73) kilogram increment in FFM, consistent with BMI being related to both lean and fat body mass components, but more-so with adiposity.

Table 3 shows correlations of anthropometric measurements with body composition, including region-specific fat depots, by sex. Height was positively associated with all body composition measures in females except percent body fat; height was not associated with body composition in males. Weight was strongly associated with both fat and FFM measures in both. Neck circumference was generally more strongly associated with measures of body fatness in males than in females, perhaps because of its proximity to the trunk, and it was more modestly associated with total body FFM. Similarly, waist circumference at its minimal point and at the umbilicus, hip circumference, and SSF—all capturing parameters associated with the trunk of the body—were particularly strongly associated with body fatness measures in males compared to females, although waist circumference was the anthropometric indicator (other than body weight) most strongly associated with total body and trunk fat in females. However, in females the association of waist circumference measures with FFM was stronger than in males, suggesting that waist circumference captured both fat and FFM depots in women while capturing the main source of fat mass in men. Arm circumference measures tended not to perform as well as other circumferential measures at capturing total body fatness, although TSF performed well at distinguishing total body, trunk, and arm fat measures from FFM, and might be recommended in women where excess fat is concentrated in extremities. Arm circumference was associated with total body FFM in males, suggesting that it was capturing more musculature in males than females. Arm muscle area was not associated with measures of fatness in either males or females, although its association with FFM was also weak.

**Table 3:** Body composition by anthropometry relative to DXA in adult females (n = 9) and males (n = 11) with achondroplasia^1^

Measurement		Fat									FFM	
	Total, kg		Percent Body Fat (%)		Trunk, kg		Total arm, kg		Total leg, kg		Lean + BMC, kg	
	F	M	F	M	F	M	F	M	F	M	F	M
Height	0.7790*	-0.1381	0.5936	-0.1621	0.7671*	-0.0961	0.7428*	-0.1463	0.7278*	-0.2085	0.7884†	0.2073
Weight	0.9545‡	0.9422§	0.7919*	0.8669‡	0.9507‡	0.9096‡	0.8341†	0.9319§	0.9073‡	0.9413§	0.8852†	0.8228†
Neck circ	0.8092†	0.9334§	0.7342*	0.8860‡	0.8221†	0.9299§	0.8858†	0.8761‡	0.6551	0.8564‡	0.6215	0.6250*
Waist circ	0.9125‡	0.9725§	0.7447*	0.9342§	0.9332‡	0.9719§	0.8308†	0.9211‡	0.8032†	0.9107‡	0.8430†	0.6524*
Umbilicus circ	0.8933†	0.9820§	0.7245*	0.9381§	0.8972†	0.9767§	0.7714*	0.9351§	0.8398†	0.9314§	0.8240†	0.6632*
Hip circ	0.8693†	0.9160‡	0.7716*	0.8469†	0.8419†	0.8678‡	0.6948*	0.9229‡	0.9106‡	0.9383§	0.7333*	0.7553†
SSF	0.4861	0.9723§	0.4666	0.9334‡	0.5072	0.9733§	0.6876	0.9329‡	0.3077	0.9469§	0.4004	0.5544
arm circ	0.7523*	0.8419†	0.6799*	0.7817†	0.7721*	0.7922†	0.7978*	0.8537‡	0.5982	0.8767‡	0.6769*	0.8586‡
TSF	0.8877†	0.8679‡	0.9054‡	0.8294†	0.8863†	0.7938†	0.8789†	0.9206‡	0.7901*	0.9332§	0.4969	0.6243*
AFA	0.8690†	0.8845‡	0.8583†	0.8354†	0.8787†	0.8087†	0.8919†	0.9328§	0.7349*	0.9570§	0.5770	0.6784*
AMA	0.2358	0.3896	0.1132	0.3323	0.2700	0.3957	0.3256	0.3436	0.0902	0.3738	0.5204	0.6988*

*DXA* dual energy x-ray absorptiometry, *FFM* fat free mass, *SSF* subscapular skinfold, *TSF* triceps skinfold, *AFA* arm fat area, *AMA* arm muscle area

^1^By Pearson correlation: *P < 0.05 to P = 0.01; †P < 0.01 to P = 0.001; ‡P < 0.001 to P = 0.0001; § P < 0.0001

## Discussion

In this pilot study, we demonstrated the feasibility of conducting a variety of measurements to capture body composition and resting energy expenditure as a foundation for conducting more extensive measurements among the achondroplasia population, with the hope of providing guidance on body composition and anthropometric measures associated with health outcomes. Although small in sample size, this study provides the first evidence that we are aware of to suggest that BIA, a straightforward measurement to obtain, could perform as well or better than anthropometry, including BMI, at estimating whole body composition parameters of total fat, percent body fat, and FFM. In this study, DXA was considered a gold standard, which allowed for partitioning of body composition parameters by body region, and sex-specific differences in body fat depots might be more readily ascertained by site-specific anthropometric measurements. Finally, REE was found to be related to FFM, consistent with benefits of enhancing the FFM component of the body to enhance the energy utilized in a resting state.

For whole body fat and FFM assessment, tetrapolar BIA performed well compared to DXA as a gold standard for predicting total fat, percent fat, and FFM in the participants with achondroplasia, even when using equations developed for the average stature population. This finding was welcome but surprising given the different body morphology of this population than those with average stature in whom the equations were derived. Derivation in a different population group could have rendered the prediction equations inaccurate for achondroplasia [17, 18, 28], and achondroplasia-specific equations derived from a larger study might ultimately be preferred. Nonetheless, our data suggest that BIA could become an important tool for evaluating and monitoring body composition in this population, and for relating it to health outcomes of interest. To our knowledge, this is the first application of BIA in persons with achondroplasia. More data are needed, however, and caution is warranted if applying other types of BIA instrumentation (hand-to-hand, foot-to-foot): for some instrumentation equations are proprietary and could not be rigorously tested, and the utility of the equations by Sun et al. [26] that account for sex, weight, and height might be dependent on the positioning of the electrodes at the hand and feet, representing the bulk of the length of the entire body.

Although BMI was strongly associated with body fatness, and only modestly associated with FFM, its association with adiposity differed by sex and was more variable than BIA-derived measures. We and others have cautioned against the use of BMI in achondroplasia given that weight is expressed against a measure of height, and the preponderance of trunk size relative to extremities is extreme in achondroplasia relative to the average stature population [16, 29, 30]. The use of BMI to characterize obesity is common, but regardless of body type it should be appreciated that BMI is not a direct measure of adiposity and should therefore not be used to infer health risk; rather, more comprehensive information on body composition and metabolic health is required to distinguish metabolically healthy and abnormal phenotypes regardless of BMI, even among the average stature population [31]. This movement beyond BMI is even more important for those with short stature conditions.

Both BIA-derived measures and BMI showed sex-related differences in whole body measures of fatness, with greater overall adiposity among women, but neither can distinguish regional fat depots. Conversely, both DXA and anthropometry captured the dramatic distinction between fat depots in the extremities in men and women. These distinctions are not unique to the population with achondroplasia, as men typically carry more fat in the abdominal region, while women tend to accumulate fat around the hips, legs, and extremities [32, 33]. These differences could be exaggerated in achondroplasia given that tissue mass is accumulated on a smaller skeletal frame. Adiposity accumulated on the hips and thighs is considered to confer less metabolic risk than are abdominal visceral fat deposits [33]. Despite concerns of excess abdominal adiposity, neither Owen et al. [30] nor Fredwall et al. [14, 15] found evidence of excess dyslipidemia or diabetes risk in studies examining body composition in achondroplasia. Conversely, high blood pressure is common and associated with body weight [13–15]. We showed here that trunk (i.e., largely abdominal) fatness contributes the most to total body fat mass in both sexes. However, Fredwell et al. found higher than expected intra-muscular fat in the extremities among adult males and females with achondroplasia [14, 15], which was linked to spinal stenosis and poorer mobility [19], suggesting health consequences of non-visceral fat depots as well. Intra-muscular fat is not detectable with DXA, but those findings suggest that attention to specific fat depots is warranted in the population with achondroplasia.

Sex differences were also observed in anthropometric measures. That height in females was positively associated with all body composition measures, except percent body fat, might be explained by the finding that excess weight in females was accumulated on the extremities if leg rather than trunk length accounts for greater variability in total height by sex, although we did not measure this. That neck circumference, waist circumference, hip circumference, and subscapular skinfolds were more strongly associated with measures of body fatness in males could be due to their proximity to the trunk, which was where more fat was accumulated in males. Conversely, in females the association of waist circumference with FFM suggests that waist circumference captured both fat and FFM depots in women, whose excess adiposity was concentrated elsewhere. Triceps skinfolds performed well at distinguishing total body, trunk, and arm fat measures from FFM, and might have utility in women since excess fat is concentrated in extremities, although it is challenging to measure. Arm circumference likely captured more musculature in males than females, given that it encircles both fat and lean mass, with lean mass tending to be higher in males. Arm muscle area was not associated with measures of fatness in either males or females, and its association with FFM was also weak. Given the difficulties in assessing arm circumference, it likely has little utility as a common measure of body composition in the achondroplasia population.

Comparisons of our data with other body composition results among adults with achondroplasia and in the average stature population suggest consistency with secular trends toward increasing overweight and obesity over the past several decades. We found percent body fat averaging over 40% in females and ~ 25% in males (with mean FFM of ~ 35 kg, fat mass ~ 25 kg in females and ~ 13 kg in males). Percent body fat among n = 27 participants with achondroplasia in 1990 using densitometry (underwater weighing) averaged ~ 28% in women and ~ 21% in men in participants averaging about 5 years younger and males 0.010 m taller than in our study, with ~ 13 kg fat in both sexes and FFM similar to what we reported [30]. More recently, among n = 10 men with achondroplasia in the UK with body composition assessed by DXA, percent body fat was 29%, higher than in average stature controls (with body fat ~ 18 and FFM ~ 41 kg, again in participants ~ 0.010 m taller than in the present study) [34]. A recent summary of body composition from the U.S. National Health and Nutrition Examination Survey (NHANES) using DXA measurements obtained from 1999 to 2006 showed ~ 38% body fat among US females and ~ 27% body fat among US males [35], akin to our findings in the achondroplasia group, compared to NHANES data collected from 1988 to 1994, where percent body fat by BIA averaged ~ 33% and ~ 24% among white females and males, respectively [36].

Despite differences in body composition, we found no differences by sex in REE, at ~ 1250 kcal/d, nor did we find REE to be associated with body weight, although it was positively associated with metabolically active FFM. Owen et al. previously found REE to be ~ 1200 kcal/d among women and 1500 kcal/d in men with achondroplasia [30]. Madsen et al. more recently found REE of 1110 kcal/d among adult females and 1416 kcals/d among adult males with achondroplasia in Norway [37]. Both of those studies showed greater height differentials between men and women, a proxy for greater FFM. In the Owen et al. study, REE was strongly positively correlated with both total weight and FFM, albeit more strongly with FFM [30]. Associations of REE with total body weight and FFM are commonly observed [24, 38], with an estimated REE of ~ 24 kcal/kg/d among average stature adults [24], similar to what we observed. The slope of the association we observed between FFM and REE was not as great in this population as observed in average stature (REE = 21.7xFFM + 374) [38] or in a reanalysis of the data published by Owen et al. (REE = 23.9xFFM + 394) [30], but average REE per unit FFM was similar in our study (36 kcal/kg) to Owen et al. (34 kcal/kg) [30]. Among Belgian average stature women across BMI categories indicative of normal adiposity to morbid obesity, REE/FFM varied from ~ 30–35 kcal/kg FFM, with mean REE over 1600 kcal/d [39]. The comparability of REE in the group with achondroplasia with average stature relative to body weight and composition suggests that REE is appropriate per unit body mass, such that low REE does not seem to account for excess undesired weight in this population. To fully define energy balance in adults with achondroplasia, a better understanding of the contribution of physical activity to energy expenditure is needed in those with achondroplasia. Data suggest higher energy expenditure associated with common ambulatory activities in individuals with achondroplasia versus average stature, likely related to differences in gait [40]. Yet, we showed that overall physical activity was low in this group of adults with achondroplasia [20], and accurately assessing fitness and physical activity remains a challenge in this population group [41]. An appreciation for conditions that can undermine regular physical activity in achondroplasia [9], and their prevention or mitigation, is warranted as part of routine care to optimize weight control.

Despite adding to the literature on assessing status of those with achondroplasia, we urge caution and encourage replication of our findings given the small sample size of this study, such that we cannot assume participant characteristics are necessarily broadly generalizable and that lack of statistical significance of some comparisons by sex or by comparison of methods are not due to being underpowered. Although conducted under rigorous conditions, we cannot assume that even the most carefully conducted measurements (for example, DXA and anthropometry) are without measurement bias given a population with a unique body morphology.

## Conclusions

These results provide an initial look at assessment techniques and associations among body composition measures. They suggest that excess weight among typical adults with achondroplasia mirrors obesity trends in the broader population, and that BIA and some key anthropometric indicators might be useful in assessing risk of excess adiposity. Our findings add to a toolkit of approaches that can improve assessment of persons with achondroplasia, and our results and those from a growing body of researchers suggest that resting energy expenditure is appropriate for body size, and that weight control might therefore be dependent on improved fitness and activity levels among this population. However, comprehensive assessment of body composition in relation to health outcomes in a larger achondroplasia cohort is required to determine associations of body composition and energy expenditure parameters with metabolic outcomes and other medical conditions.

## Supplementary Information

Supplementary Material 1

## Data Availability

Datasets generated or analysed during this study are available from the corresponding author on reasonable request.

## Abbreviations

DXA: Dual energy X-ray absorptiometry
BIA: Bioelectrical impedance analysis
REE: Resting energy expenditure
FFM: Fat free mass
BMI: Body mass index
MRI: Magnetic resonance imaging
JHH: Johns Hopkins Hospital
AFA: Arm fat area
AMA: Arm muscle area
LPA: Little People of America
NHANES: National Health and Nutrition Examination Survey
