# Supplementary material for: Body composition, anthropometry, and resting energy expenditure in adults with achondroplasia: a pilot study to determine best practices
Source: Orphanet J Rare Dis. 2025 Oct 21;20:527. doi: 10.1186/s13023-025-03912-z (PMC12542460; doi:10.1186/s13023-025-03912-z)
Supplement: Supplementary file 1 — Supplementary Material 1 [file 13023_2025_3912_MOESM1_ESM.docx]

Additional File

Additional Figure 1. Bland-Altman Plot for Total Body Fat by BIA vs DXA, with output

. blandaltman totalfat_bia_kg totalfat_dxa_kg, plot(difference)

A: totalfat_bia_kg

B: totalfat_dxa_kg

DIFFERENCES...

Calculation N Mean SD Interval(s)

A-B 20 -.6414796 1.818178

. regress difference mean

Source | SS df MS Number of obs = 20

-------------+---------------------------------- F(1, 18) = 0.02

Model | .083317229 1 .083317229 Prob > F = 0.8788

Residual | 62.7263703 18 3.48479835 R-squared = 0.0013

-------------+---------------------------------- Adj R-squared = -0.0542

Total | 62.8096876 19 3.30577303 Root MSE = 1.8668

------------------------------------------------------------------------------

__000002 | Coefficient Std. err. t P>|t| [95% conf. interval]

-------------+----------------------------------------------------------------

__000003 | -.0068291 .0441659 -0.15 0.879 -.0996182 .08596

_cons | -.5129503 .9301565 -0.55 0.588 -2.467137 1.441236

------------------------------------------------------------------------------

-> regression-based bias: -.5129503 + -.0068291 × Mean(A,B)

. regress adj_abs_resid mean

Source | SS df MS Number of obs = 20

-------------+---------------------------------- F(1, 18) = 0.12

Model | .245000288 1 .245000288 Prob > F = 0.7302

Residual | 35.9504534 18 1.99724741 R-squared = 0.0068

-------------+---------------------------------- Adj R-squared = -0.0484

Total | 36.1954537 19 1.90502388 Root MSE = 1.4132

------------------------------------------------------------------------------

__00000C | Coefficient Std. err. t P>|t| [95% conf. interval]

-------------+----------------------------------------------------------------

__000003 | .0117107 .033436 0.35 0.730 -.0585357 .0819571

_cons | 1.545027 .7041791 2.19 0.042 .0656017 3.024453

------------------------------------------------------------------------------

-> regression-based SD: 1.545027 + .0117107 × Mean(A,B)

-> regression-based 95% LLOA: -3.541148 + -.0297816 × Mean(A,B)

-> regression-based 95% ULOA: 2.515247 + .0161233 × Mean(A,B)

Supplementary Figure 2. Bland-Altman Plot for Percent Body Fat by BIA vs DXA, with output

. blandaltman fatpercent_bia fatpercent_dxa, plot(difference)

A: fatpercent_bia

B: fatpercent_dxa Total Fat %

DIFFERENCES...

Calculation N Mean SD Interval(s)

A-B 20 -1.525 3.362267

. regress difference mean

Source | SS df MS Number of obs = 20

-------------+---------------------------------- F(1, 18) = 0.00

Model | .037243269 1 .037243269 Prob > F = 0.9561

Residual | 214.754647 18 11.9308137 R-squared = 0.0002

-------------+---------------------------------- Adj R-squared = -0.0554

Total | 214.79189 19 11.3048363 Root MSE = 3.4541

------------------------------------------------------------------------------

__000002 | Coefficient Std. err. t P>|t| [95% conf. interval]

-------------+----------------------------------------------------------------

__000003 | -.0036595 .0654995 -0.06 0.956 -.1412689 .1339498

_cons | -1.406733 2.253286 -0.62 0.540 -6.14071 3.327245

------------------------------------------------------------------------------

-> regression-based bias: -1.406733 + -.0036595 × Mean(A,B)

. regress adj_abs_resid mean

Source | SS df MS Number of obs = 20

-------------+---------------------------------- F(1, 18) = 0.39

Model | 2.40761053 1 2.40761053 Prob > F = 0.5421

Residual | 112.224098 18 6.23467212 R-squared = 0.0210

-------------+---------------------------------- Adj R-squared = -0.0334

Total | 114.631709 19 6.03324783 Root MSE = 2.4969

------------------------------------------------------------------------------

__00000C | Coefficient Std. err. t P>|t| [95% conf. interval]

-------------+----------------------------------------------------------------

__000003 | -.0294236 .0473489 -0.62 0.542 -.1288999 .0700527

_cons | 4.287843 1.628876 2.63 0.017 .8657013 7.709985

------------------------------------------------------------------------------

-> regression-based SD: 4.287843 + -.0294236 × Mean(A,B)

-> regression-based 95% LLOA: -9.810751 + .0540097 × Mean(A,B)

-> regression-based 95% ULOA: 6.997285 + -.0613288 × Mean(A,B)

Supplementary Figure 3. Bland-Altman Plot for FFM by BIA vs DXA, with output

. blandaltman ffm_bia_kg totallean_dxa_kg, plot(difference)

A: ffm_bia_kg

B: totallean_dxa_kg

DIFFERENCES...

Calculation N Mean SD Interval(s)

A-B 20 1.209205 1.815829

. regress difference mean

Source | SS df MS Number of obs = 20

-------------+---------------------------------- F(1, 18) = 0.00

Model | .000812641 1 .000812641 Prob > F = 0.9880

Residual | 62.6466679 18 3.48037044 R-squared = 0.0000

-------------+---------------------------------- Adj R-squared = -0.0555

Total | 62.6474806 19 3.29723582 Root MSE = 1.8656

------------------------------------------------------------------------------

__000002 | Coefficient Std. err. t P>|t| [95% conf. interval]

-------------+----------------------------------------------------------------

__000003 | -.001373 .0898554 -0.02 0.988 -.1901523 .1874063

_cons | 1.258655 3.262955 0.39 0.704 -5.596559 8.11387

------------------------------------------------------------------------------

-> regression-based bias: 1.258655 + -.001373 × Mean(A,B)

. regress adj_abs_resid mean

Source | SS df MS Number of obs = 20

-------------+---------------------------------- F(1, 18) = 0.05

Model | .086183243 1 .086183243 Prob > F = 0.8220

Residual | 29.774634 18 1.65414633 R-squared = 0.0029

-------------+---------------------------------- Adj R-squared = -0.0525

Total | 29.8608172 19 1.57162196 Root MSE = 1.2861

------------------------------------------------------------------------------

__00000C | Coefficient Std. err. t P>|t| [95% conf. interval]

-------------+----------------------------------------------------------------

__000003 | -.0141398 .0619468 -0.23 0.822 -.1442851 .1160055

_cons | 2.360525 2.249497 1.05 0.308 -2.365492 7.086542

------------------------------------------------------------------------------

-> regression-based SD: 2.360525 + -.0141398 × Mean(A,B)

-> regression-based 95% LLOA: -3.367888 + .0263405 × Mean(A,B)

-> regression-based 95% ULOA: 5.885199 + -.0290865 × Mean(A,B)
